# Subphrenic Lymph Node Metastasis Predicts Poorer Prognosis for Nasopharyngeal Carcinoma Patients With Metachronous Metastasis

**DOI:** 10.3389/fonc.2021.726179

**Published:** 2021-10-01

**Authors:** Xue-Fang Zhang, Yan Zhang, Xu-Wei Liang, Jia-Luo Chen, Sheng-Fang Zhi, Wen-Jing Yin, Meng-Yao Wang, En-Lai Dong, Dong-Ping Chen

**Affiliations:** ^1^ Radiotherapy Department, Affiliated Dongguan Hospital, Southern Medical University, Dongguan, China; ^2^ Nuclear Medicine Department, Affiliated Dongguan Hospital, Southern Medical University, Dongguan, China; ^3^ Department of Radiation Oncology, Affiliated Cancer Hospital and Institute of Guangzhou Medical University, Guangzhou, China

**Keywords:** nasopharyngeal carcinoma (NCP), metachronous metastasis, subphrenic lymph node metastasis, distant lymph node metastasis, prognosis

## Abstract

**Aim:**

We retrospectively analyzed the distribution of distant lymph node metastasis and its impact on prognosis in patients with metastatic NPC after treatment.

**Methods:**

From 2010 to 2016, 219 NPC patients out of 1,601 (182 from the Affiliated Cancer Hospital and Institute of Guangzhou Medical University, and 37 from the Affiliated Dongguan Hospital, Southern Medical University) developed distant metastasis after primary radiation therapy. Metastatic lesions were divided into groups according to location: bones above the diaphragm (supraphrenic bone, SUP-B); bones below the diaphragm (subphrenic bone, SUB-B); distant lymph nodes above the diaphragm (supraphrenic distant lymph nodes, SUP-DLN); distant lymph nodes below the diaphragm (subphrenic distant lymph nodes, SUB-DLN), liver, lung, and other lesions beyond bone/lung/distant lymph node above the diaphragm (supraphrenic other lesions, SUP-OL); other lesions beyond bone/liver/distant lymph node below the diaphragm (subphrenic other lesions, SUB-OL); the subtotal above the diaphragm (supraphrenic total lesions, SUP-TL); and the subtotal below the diaphragm (subphrenic total lesions, SUB-TL). Kaplan–Meier methods were used to estimate the probability of patients’ overall survival (OS). Univariate and multivariate analyses were applied using the Cox proportional hazard model to explore prediction factors of OS.

**Results:**

The most frequent metastatic locations were bone (45.2%), lung (40.6%), liver (32.0%), and distant lymph nodes (20.1%). The total number of distant lymph node metastasis was 44, of which 22 (10.0%) were above the diaphragm, 18 (8.2%) were below the diaphragm, and 4 (1.8%) were both above and below the diaphragm. Age (HR: 1.02, 95% CI: 1.00, 1.03, p = 0.012), N stage (HR: 1.26, 95% CI: 1.04, 1.54, p = 0.019), number of metastatic locations (HR: 1.39, 95% CI: 1.12, 1.73, p = 0.003), bone (HR: 1.65, 95% CI: 1.20, 2.25, p = 0.002), SUB-B (HR: 1.51, 95% CI: 1.07, 2.12, p = 0.019), SUB-DLN (HR: 1.72, 95% CI: 1.03, 2.86, p = 0.038), and SUB-O L(HR: 4.46, 95% CI: 1.39, 14.3, p = 0.012) were associated with OS. Multivariate analyses revealed that a higher N stage (HR: 1.23, 95% CI: 1.00, 1.50, p = 0.048), SUB-DLN (HR: 1.72, 95% CI: 1.02, 2.90, p = 0.043), and SUB-OL (HR: 3.72, 95% CI: 1.14, 12.16, p = 0.029) were associated with worse OS.

**Conclusion:**

Subphrenic lymph node metastasis predicts poorer prognosis for NPC patients with metachronous metastasis; however, this needs validation by large prospective studies.

## Introduction

Nasopharyngeal carcinoma (NPC) is one of the most common malignant tumors in South China with 18%–50% of treatment failure due to distant metastasis (1). As there are many lymphatic capillaries in the mucosa of the nasopharynx, NPC is prone to lymph node metastasis, with cervical lymph node involvement as high as 85%–90% in newly diagnosed NPC patients (2–4). Tumor cells use the lymphatic duct and lymph nodes for metastasis and the colonization of peripheral organs (5).

Brown et al. confirmed that cancer cells not only pass through the sentinel lymph nodes and then enter the lymphatic duct to metastasize to distant organs but also directly enter the bloodstream through blood vessels in the lymph nodes (6). Ethel R. Pereira et al. found that isolated cancer cells in the lymph nodes were located within 5 mm of blood vessels and that mice with complete lymph nodes had more circulating tumor cells and lung metastasis than those that underwent lymph node resection (7), proving that lymph node metastasis is diffused from lymph nodes invading the blood vessels rather than by exporting lymph vessels. This poses an important question: does the location of distant lymph node NPC metastasis impact prognosis, and if so, how?

In this paper, we retrospectively analyzed the distribution of distant lymph node metastasis and its impact on prognosis in patients with metastatic NPC after treatment. We hope that the study of tumor characteristics according to the location and route of metastasis will help to determine the biological explanation of tumor behavior, explain the related survival results, and guide disease monitoring and treatment selection.

## Materials and Methods

### Patients

In this retrospective study, 1,601 NPC patients from 2010 to 2016 were initially treated at the Affiliated Cancer Hospital and Institute of Guangzhou Medical University (1,214) and the Affiliated Dongguan Hospital, Southern Medical University (387). Two hundred nineteen patients (182 from the Affiliated Cancer Hospital and Institute of Guangzhou Medical University and 37 from the Affiliated Dongguan Hospital, Southern Medical University) developed distant metastasis after primary radiation therapy (RT).

This study’s inclusion criteria were i) histologically confirmed NPC and ii) radiographically detectable metastatic disease after initial radiation therapy (more than 3 months) on the basis of subsequent follow-up. The exclusion criteria were i) other malignancies and ii) HIV, tuberculosis, or other chronic inflammatory diseases (e.g., inflammatory bowel disease).

All clinical data were collected when metastasis was diagnosed, including magnetic resonance imaging of the head and neck regions, radiographs or computed tomography (CT) of the chest, ultrasonography or CT scans of the abdomen, and whole-body bone scans. Positron emission tomography with 2-deoxy-2-[fluorine-18]fluoro-D-glucose integrated with computed tomography(18F FDG PET/CT) were performed to confirm the metastasis of 44 patients (7 from the Affiliated Cancer Hospital and Institute of Guangzhou Medical University and 37 from the Affiliated Dongguan Hospital, Southern Medical University).

All 219 patients were treated with intensity-modulated radiation therapy (IMRT) during the initial treatment. Among them, 14 (6.4%) did not receive chemotherapy, and the other 205 (93.6%) received chemotherapy. One hundred seventy-one (78.1%) were treated with concurrent chemotherapy, including 19 (8.68%) with concurrent chemotherapy, 73 (33.3%) with neoadjuvant chemotherapy plus concurrent chemotherapy, 12 (5.5%) with concurrent chemotherapy plus adjuvant chemotherapy, and 67 (30.6%) with neoadjuvant chemotherapy plus concurrent chemotherapy plus adjuvant chemotherapy; 34 (15.5%) received non-concurrent chemotherapy, among which 26 (11.87%) received neoadjuvant chemotherapy, 1 (0.5%) received adjuvant chemotherapy, and 7 (3.2%) received neoadjuvant chemotherapy plus adjuvant chemotherapy.

After distant metastasis, cisplatin-based combination chemotherapy was recommended for most patients (209, 95.4%). Other agents were 5-fluorouracil, paclitaxel (albumin paclitaxel or paclitaxel liposomal), docetaxel, gemcitabine, cyclophosphamide, vincristine, bleomycin, capecitabine, and S-1. Supportive management with no anticancer treatment was provided for four patients (4,1.8%). Surgical resection, radiation therapy, radiofrequency ablation, and trans-arterial chemoembolization were prescribed if the doctors thought it was valuable to do so.

This study was approved by the ethics committee of the Affiliated Cancer Hospital and Institute of Guangzhou Medical University with the approval number: ZN2021-05.

### Distribution of Metastatic Lesions and Other Variables

The criteria for distant lymph node (DLN) metastasis were i) CT/MRI showing a minimum lymph node diameter of ≥10 mm; ii) central necrosis or annular reinforcement; iii) extracapsular invasion of the lymph nodes (irregular enhancement of the lymph node margin; iv) some or all of the surrounding fat spaces not visible; v) lymph nodes fused with each other; and vi) 18F FDG PET/CT: SUV of the lymph nodes higher than the abdominal aorta, and/or CT images with the above features (8).

Metastatic lesions were divided into groups according to location: bones above the diaphragm (supraphrenic bone, SUP-B); bones below the diaphragm (subphrenic bone, SUB-B); distant lymph nodes above the diaphragm (supraphrenic distant lymph nodes, SUP-DLN); distant lymph nodes below the diaphragm (subphrenic distant lymph nodes, SUB-DLN), liver, lung, other lesions above the diaphragm (supraphrenic other lesions beyond bone/lung/distant lymph node, SUP-OL); other lesions below the diaphragm (subphrenic other lesions beyond bone/liver/distant lymph node, SUB-OL); the subtotal above the diaphragm (supraphrenic total lesions, SUP-TL); and the subtotal below the diaphragm (subphrenic total lesions, SUB-TL).

SUP-TL included SUP-B, SUP-DLN, lung, and SUP-OL. SUB-TL included SUB-B, SUB-DLN, liver, and SUB-OL. SUP-B is defined as lesions of the C-spine, T-spine, ribs, sternum, scapula, humerus, and clavicle. SUB-B is defined as lesions of the L-spine, sacrum, pelvic bone, and femur. SUP-DLN is defined as mediastinal LN, axillary LN, and hilar LN (excluding neck LN). SUB-DLN is defined as retroperitoneal LN, pelvic LN, hepatic hilar LN, and inguinal LN. SUP-OL is defined as other metastases above the diaphragm and beyond the bone/lung/distant lymph node such as the pleura and thyroid. SUB-OL is defined as other metastases below the diaphragm and beyond the bone/liver/distant lymph node such as the spleen and adrenal glands.

The primary disease, NPC, was T and N staged according to the American Joint Committee on Cancer (AJCC) Cancer Staging Manual (8^th^ Edition) (9). The variables assessed in this study included sex, age, body mass index before primary therapy (BMI1), body mass index during metastasis (BMI2), the T/N/TNM staging of the primary disease NPC, disease-free interval (DFI), local recurrence, overall survival (OS), and the total organs of the metastatic lesions (organs-n).

### Follow-Up and Endpoints

Patients were routinely followed up every two cycles during systemic chemotherapy and every 2 to 3 months during no anticancer treatment until death. OS was defined as the interval between the date of distant metastasis to the date of death of any cause. DFI was defined as the interval from the date of initial diagnosis of NPC to the date of distant metastasis. Data from patients alive at the end of study (December 31, 2020) were censored. We verified survival status on August 31, 2020, by direct telecommunication with the patient or family members and by checking the clinic attendance records.

### Statistical Analysis

Continuous variables were described using mean and standard deviation (SD) for normally distributed data and median and interquartile [IQR] for non-normally distributed data. The Student t-test or Mann–Whitney U test were used for continuous variables between groups. Frequency and percentage were used to describe the categorical data, and the chi-square test to test the difference.

The Kaplan–Meier method was used to estimate the probability of patients’ OS. Survival curves were drawn to compare the difference between/among covariate groups, and the log-rank test was applied accordingly. Univariate and multivariate analyses were applied using the Cox proportional hazard model to explore prediction factors of OS. Variables with a p<0.1 in the univariate model were kept for multivariate analyses. A stepwise variable selection procedure (with iterations between the “forward” and “backward” steps) was applied to obtain the best candidate for the final Cox proportional hazards model. The chosen significance level for entry (SLE) and for stay (SLS) was 0.25. A p value <0.05 was considered statistically significant. All statistical analyses were performed using R (software version 6.3, https://www.r-project.org/).

## Results

### Patient Characteristics

The patient characteristics of metachronous metastatic NPC (n = 219) are described in **Table 1**. The mean age at diagnosis of metastatic NPC was 50.2 years (SD, 11.4). One hundred and six (48.4%) were more than 50 years old. One hundred seventy-four (79.5%) were male, and 45 (20.5%) were female. Before the first radiotherapy treatment, 32 (14.6%), 29 (13.2%), 122 (55.7%), and 36 (16.4%) were T1, T2, T3, and T4 stages, respectively. Eleven (5.0%), 91 (41.6%), 76 (34.7%), and 41 (18.7%) were N0, N1, N2, and N3 stages, respectively.

**Table 1:** Patient characteristics.

	Level	Overall	DLN (-)	DLN (+)	p
No (%)		219 (100%)	175 (79.9%)	44 (20.1%)	
Sex no. (%)	Male	174 (79.5)	142 (81.1)	32 (72.7)	0.305
	Female	45 (20.5)	33 (18.9)	12 (27.3)	
Age no. (%)	≤50	106 (48.4)	79 (45.1)	27 (61.4)	0.079
	>50	113 (51.6)	96 (54.9)	17 (38.6)	
BMI1 [median (IQR)]		21.4 [19.9, 23.5]	21.5 [20.1, 23.5]	20.6 [18.9, 23.5]	0.074
BMI2 [median (IQR)]		20.3 [18.7, 21.9]	20.3 [18.8, 21.9]	19.4 [18.2, 21.8]	0.150
T no. (%)	1	32 (14.6)	25 (14.3)	7 (15.9)	0.256
	2	29 (13.2)	20 (11.4)	9 (20.5)	
	3	122 (55.7)	98 (56.0)	24 (54.5)	
	4	36 (16.4)	32 (18.3)	4 (9.1)	
N no. (%)	0	11 (5.0)	11 (6.3)	0 (0.0)	0.311
	1	91 (41.6)	74 (42.3)	17 (38.6)	
	2	76 (34.7)	58 (33.1)	18 (40.9)	
	3	41 (18.7)	32 (18.3)	9 (20.5)	
TNM no. (%)	I	1 (0.5)	1 (0.6)	0 (0.0)	0.355
	II	29 (13.2)	20 (11.4)	9 (20.5)	
	III	119 (54.3)	95 (54.3)	24 (54.5)	
	IVa	70 (32.0)	59 (33.7)	11 (25.0)	
Recurrence no. (%)	No	175 (79.9)	137 (78.3)	38 (86.4)	0.325
	Yes	44 (20.1)	38 (21.7)	6 (13.6)	
DFI [median (IQR)]		20.1 [10.0, 33.6]	18.0 [9.3, 31.4]	28.3 [17.0, 42.8]	0.003
OS no. (%)	No	58 (26.5)	45 (25.7)	13 (29.5)	0.746
	Yes	161 (73.5)	130 (74.3)	31 (70.5)	
OS time [median (IQR)]		13.2 [7.3, 25.3]	13.7 [7.1, 26.1]	11.0 [7.7, 20.5]	0.464
Organ N [mean (SD)]		1.5 (0.7)	1.3 (0.6)	2.2 (0.8)	<0.001
Bone no. (%)	No	120 (54.8)	91 (52.0)	29 (65.9)	0.137
	Yes	99 (45.2)	84 (48.0)	15 (34.1)	
Liver no. (%)	No	149 (68.0)	121 (69.1)	28 (63.6)	0.604
	Yes	70 (32.0)	54 (30.9)	16 (36.4)	
Lung no. (%)	No	130 (59.4)	100 (57.1)	30 (68.2)	0.246
	Yes	89 (40.6)	75 (42.9)	14 (31.8)	
Other lesion no. (%)	No	199 (90.9)	162 (92.6)	37 (84.1)	0.138
	Yes	20 (9.1)	13 (7.4)	7 (15.9)	
SUP-OL no. (%)	No	202 (92.2)	165 (94.3)	37 (84.1)	0.051
	Yes	17 (7.8)	10 (5.7)	7 (15.9)	
SUB-OL no. (%)	No	216 (98.6)	173 (98.9)	43 (97.7)	0.492
	Yes	3 (1.4)	2 (1.1)	1 (2.3)	
SUP-B no. (%)	No	149 (68.0)	119 (68.0)	30 (68.2)	1.000
	Yes	70 (32.0)	56 (32.0)	14 (31.8)	
SUB-B no. (%)	No	165 (75.3)	132 (75.4)	33 (75.0)	1.000
	Yes	54 (24.7)	43 (24.6)	11 (25.0)	
SUP-TL no. (%)	No	65 (29.7)	55 (31.4)	10 (22.7)	0.356
	Yes	154 (70.3)	120 (68.6)	34 (77.3)	
SUB-TL no. (%)	No	106 (48.4)	87 (49.7)	19 (43.2)	0.501
	Yes	113 (51.6)	88 (50.3)	25 (56.8)	

DLN, distant lymph nodes; no., number; BMI1, body mass index before primary therapy; BMI2, body mass index during metastasis; T, T staging of the primary disease of NPC; N, N staging of the primary disease of NPC; TNM, TNM staging of the primary disease of NPC; DFI, disease-free interval; OS, overall survival; SUP-OL, supraphrenic other lesions; SUB-OL, subphrenic other lesions; SUP-B, supraphrenic bone; SUB-B, subphrenic bone; SUP-TL, supraphrenic total lesions; SUB-TL, subphrenic total lesions.

The median OS of metachronous metastatic NPC was 13.2 months (IQR, 7.3, 25.3), and the median DFI was 20.1 months (IQR, 10.0, 33.6). The most frequent metastatic locations were bone (99/219, 45.2%), lung (89/219, 40.6%), liver (70/219, 32.0%), and distant lymph nodes (44/219, 20.1%). There were 142 (64.8%), 52 (23.7%), 24 (11.0%), and 1 (0.5%) patients with one, two, three, and four metastatic locations, respectively.

### Distribution of Distant Lymph Node Metastasis

The distribution of distant lymph node metastasis is described in **Table 2**. The total number of distant lymph node metastasis was 44/219 (20.1%), of which 22/219 (10.0%) were above the diaphragm, 18/219 (8.2%) were below the diaphragm, and 4/219 (1.8%) were both above and below the diaphragm. The median number of distant lymph node metastasis in 44 patients was 3 (range, 1–7), 10 (22.7%) had one DLN metastasis, 24 (54.5%) had two to four DLN metastases, and 10 (22.7%) had five or more than five DLN metastases.

**Table 2:** The distribution of distant lymph node metastasis.

Site	No. (%)
SUP-DLN	22 (10.0%)
SUB-DLN	18 (8.2%)
SUP-LN and SUB-LN	4 (1.8%)
Mediastinal LN	18 (8.2%)
Axillary LN	8 (3.7%)
Hilar LN	3 (1.4%)
Internal mammary lymph nodes	2 (0.9%)
Retroperitoneal LN	20 (9.1%)
Pelvic LN	1 (0.4%)
Hepatic hilar LN	1 (0.4%)
Inguinal LN	2 (0.8%)

No., number; SUP-DLN, supraphrenic distant lymph node; SUB-DLN, subphrenic distant lymph node.

Distant lymph node metastases above the diaphragm were located in the mediastinal LN (18/219, 8.2%), axillary LN (8/219, 3.7%), hilar LN (3/219, 1.4%), and the internal mammary lymph nodes (2/219, 0.9%). For distant lymph node metastasis below the diaphragm, there were 20/219 (10.0%) in the retroperitoneal LN, 1/219 (0.4%) in the pelvic LN, 1/219 (0.4%) in the hepatic hilar LN, and 2/219 (0.9%) in the inguinal LN.

### Distant Lymph Node Metastasis Below the Diaphragm Indicates Poorer Prognosis

**Figure 1:**
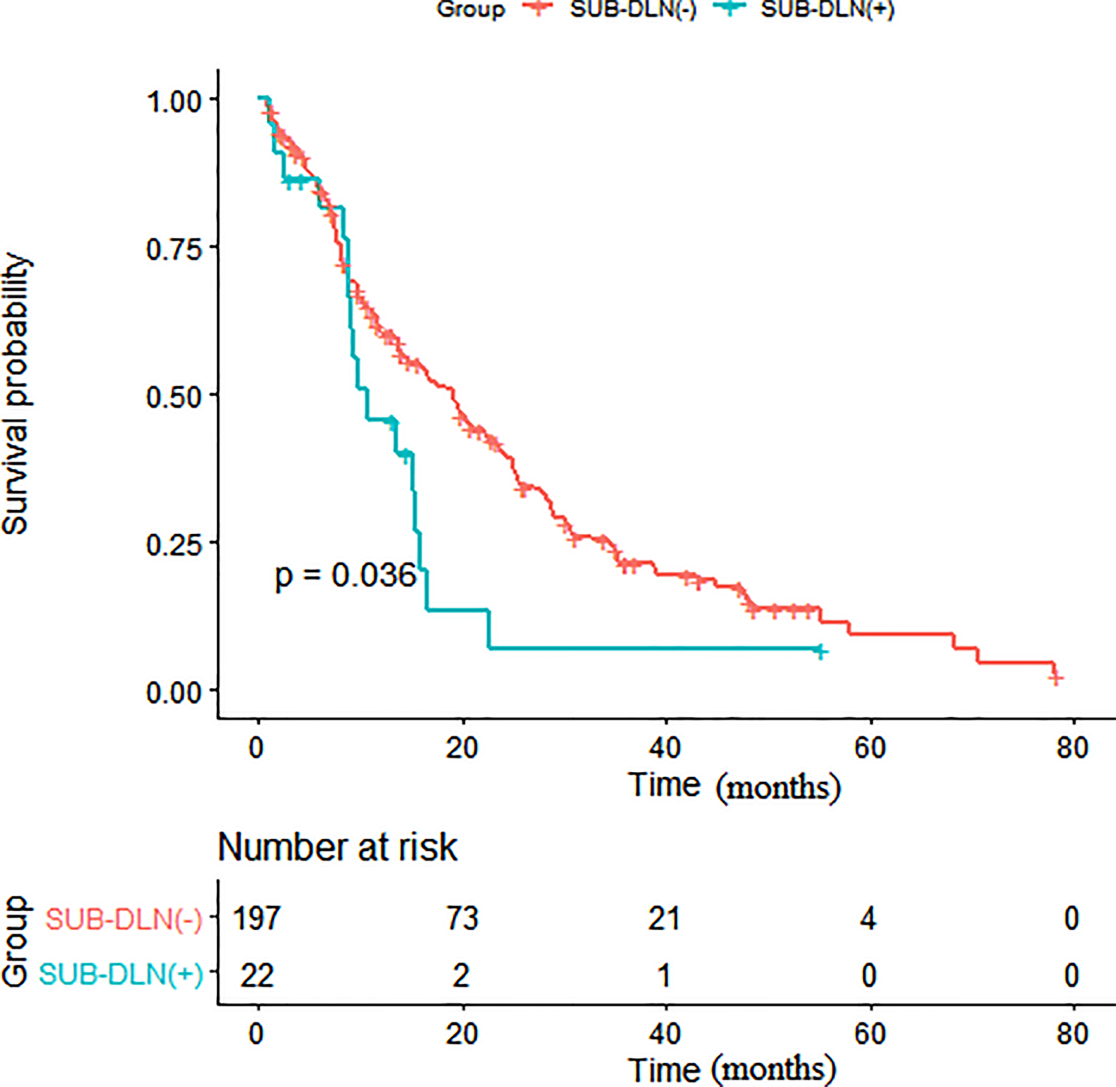
Kaplan-Meier survival analysis of patients with and without SUB-DLN. (SUB-DLN, subphrenic distant lymph node).

Survival analysis showed that patients with distant lymph node metastasis below the diaphragm had poorer OS than those without distant lymph node metastasis below the diaphragm (p = 0.036) (**Figure 1**). As shown in **Table 3**, in the univariate analysis, age (HR: 1.02, 95% CI: 1.00, 1.03, p = 0.012), a higher N stage of the primary disease NPC (HR: 1.26, 95% CI: 1.04, 1.54, p = 0.019), more metastatic locations (HR: 1.39, 95% CI: 1.12, 1.73, p = 0.003), bone (HR: 1.65, 95% CI: 1.20, 2.25, p = 0.002), SUB-B (HR: 1.51, 95% CI: 1.07, 2.12, p = 0.019), SUB-DLN (HR: 1.72, 95% CI: 1.03, 2.86, p = 0.038), and SUB-OL (HR: 4.46, 95% CI: 1.39, 14.3, p = 0.012) were associated with worse OS.

**Table 3:** Univariate analysis and multivariate analyses of clinicopathologic characteristics.

Characteristic	Univariate analysis			Multivariate analyses		
	HR	95% CI	p-value	HR	95% CI	p-value
Sex	0.88	0.59, 1.30	0.521			
Age	1.02	1.00, 1.03	0.012			
BMI1	0.99	0.94, 1.04	0.629			
BMI2	0.98	0.92, 1.04	0.451			
T	0.96	0.80, 1.15	0.612			
N	1.26	1.04, 1.54	0.019	1.23	1.00,1.50	0.048
TNM	1.22	0.95, 1.56	0.110			
Recurrence	1.02	0.68, 1.53	0.928			
DFI	1.00	0.99,1.01	0.855			
Organ_n	1.39	1.12, 1.73	0.003			
Bone	1.65	1.20,2.25	0.002			
SUP-B	1.27	0.92, 1.75	0.146			
SUB-B	1.51	1.07, 2.12	0.019	1.36	0.96,1.93	0.083
Liver	1.20	0.86, 1.67	0.281			
Lung	0.92	0.67, 1.27	0.624			
SUP-OL	1.03	0.57, 1.85	0.933			
SUB-OL	4.46	1.39, 14.3	0.012	3.72	1.14, 12.16	0.029
DLN	1.09	0.73,1.61	0.674			
DLN_n▲	1.08	0.89,1.30	0.459			
SUP-DLN	0.89	0.54, 1.46	0.643			
SUB-DLN	1.72	1.03, 2.86	0.038	1.72	1.02, 2.90	0.043
SUP-TL	1.04	0.73, 1.48	0.828			
SUB-TL	1.30	0.95, 1.77	0.104			

▲DLN_n:1/2-4/≥5.

HR, hazard ratio; CI, confidence interval; BMI1, body mass index before primary therapy; BMI2, body mass index during metastasis; T, T staging of the primary disease of NPC; N, N staging of the primary disease of NPC; TNM, TNM staging of the primary disease of NPC; DFI, disease-free interval; Organ_n, number of metastatic locations; SUP-B, supraphrenic bone; SUB-B, subphrenic bone; SUP-OL, supraphrenic other lesions; SUB-OL, subphrenic other lesions; DLN, distant lymph node; DLN_n, number of distant lymph node; SUP-DLN, supraphrenic distant lymph node; SUB-DLN, subphrenic distant lymph node; SUP-TL, supraphrenic total lesions; SUB-TL, subphrenic total lesions.

**Figure 2:**
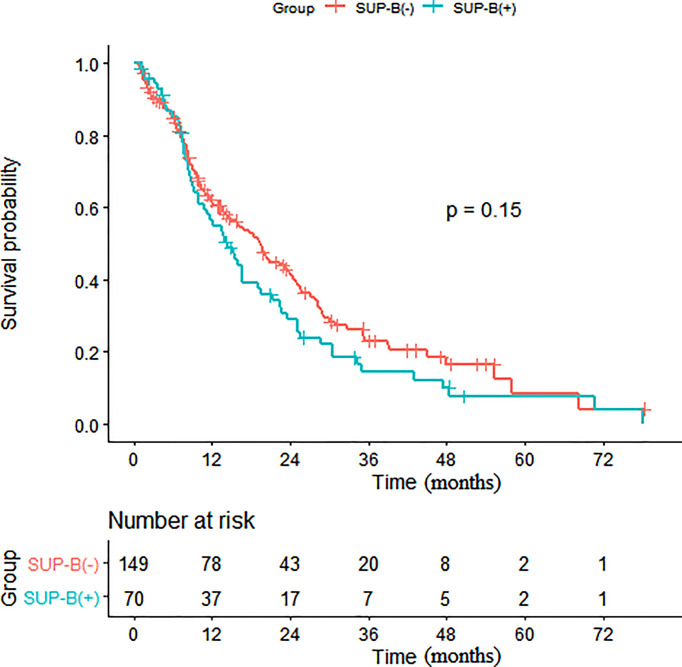
Kaplan-Meier survival analysis of patients with and without SUP-B. (SUP-B, supraphrenic bone).

**Figure 3:**
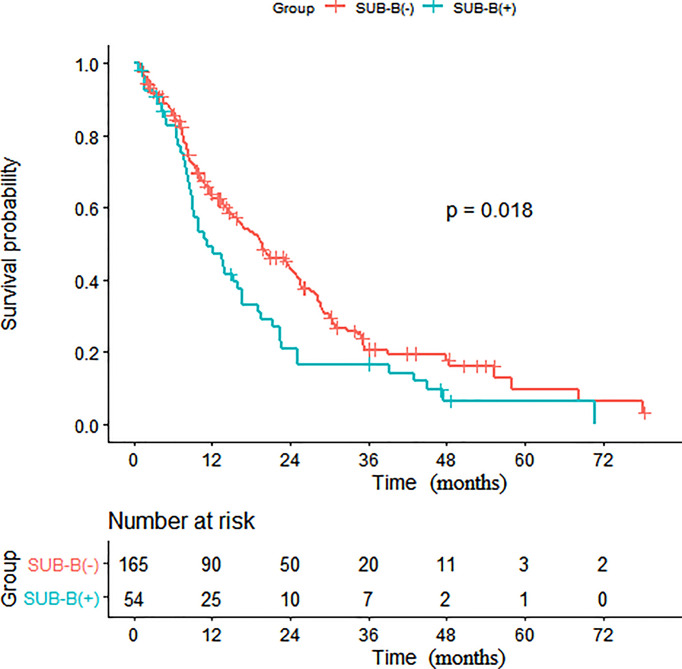
Kaplan-Meier survival analysis of patients with and without SUB-B. (SUB-B, subphrenic bone).

**Figure 4:**
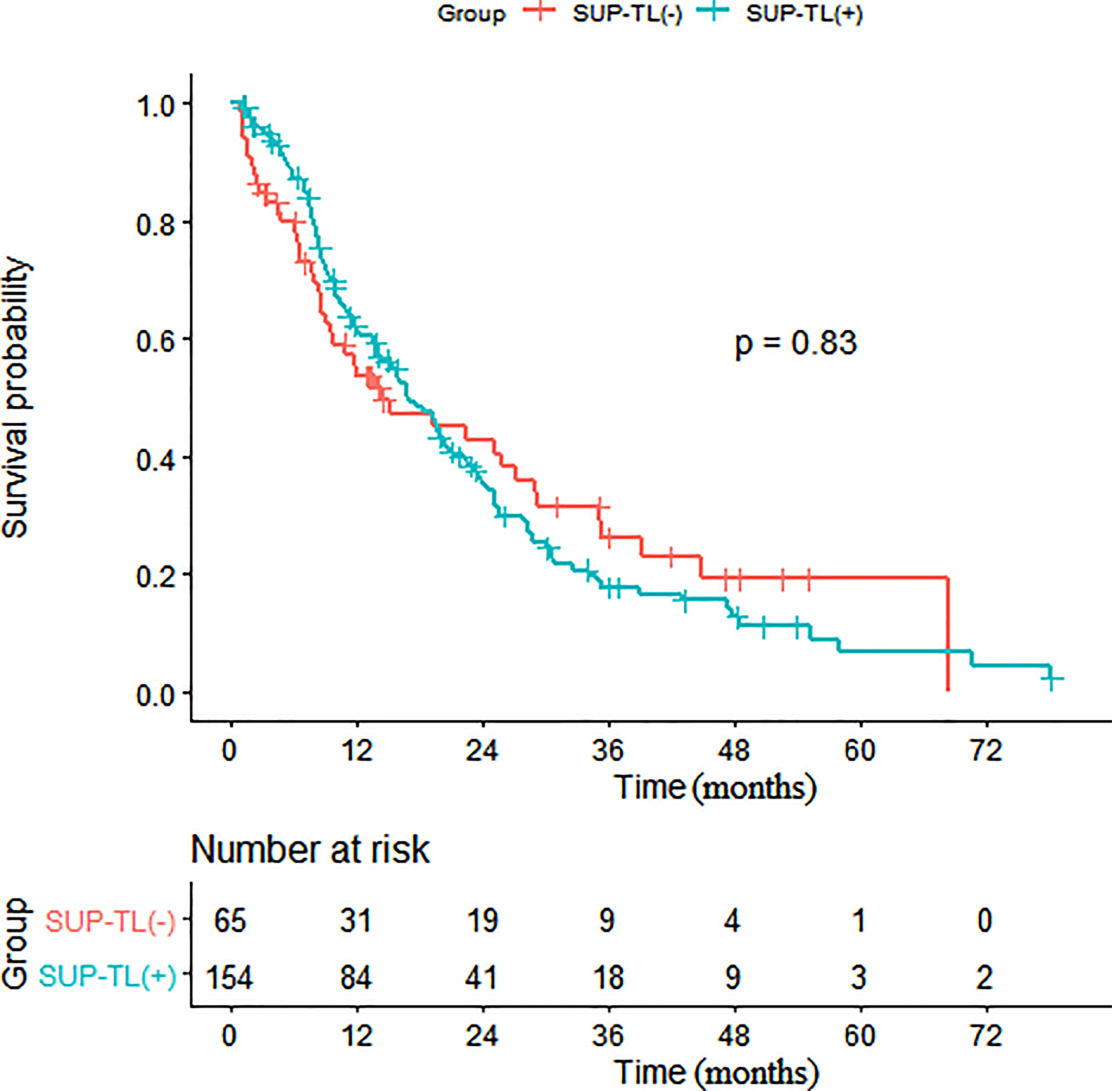
Kaplan-Meier survival analysis of patients with and without SUP-TL. (SUP-TL, supraphrenic total lesions).

Subsequent multivariate analyses revealed that a higher N stage (HR: 1.23, 95% CI: 1.00, 1.50, p = 0.048), SUB-DLN (HR: 1.72, 95% CI: 1.02, 2.90, p = 0.043), and SUB-OL (HR: 3.72, 95% CI: 1.14, 12.16, p = 0.029) were associated with worse OS. Kaplan–Meier survival analysis of patients with and without SUB-DLN is shown in **Figure 1**. Kaplan–Meier survival analysis of patients with and without SUP-B/SUB-B/SUB-TL is shown in **Figures 2**–**4**.

## Discussion

Lymph nodes are the central transport center of circulating immune cells, with the lymphatic drainage system of the body a coherent whole separated by particular anatomical boundaries (10–12). Patients with extra-regional lymph node metastasis are considered to have better prognosis than those with solid organ metastasis among some metastatic malignancies. The research by Hong Pan showed that patients with distant lymph node metastasis (DLNM) had similar breast cancer–specific survival (BCSS) and OS as those with ipsilateral supraclavicular lymph node metastasis (ISLM), whereas those with distant metastasis (not DLNM) had significantly poorer BCSS and OS (12). Similarly, Francesca Magnoni found that although contralateral axillary lymph node metastasis after treatment belongs to distant metastasis (distant lymph node metastasis), its OS is significantly better than the distant metastasis of other organs (13). Yuki Mukai also found that cervical cancer without metastasis of other organs but with distant lymph node metastasis (supraclavicular/mesentery/mediastinum lymph node) had a good local control rate. The 2-year overall, cancer-specific, and progression-free survival as well as the local control of primary tumor rates were 51.3%, 51.3%, 46.9%, and 67.9%, respectively (14).

The lymphatic drainage routes of the body’s organs are local lymph nodes, retroperitoneal lymph nodes, the thoracic duct, and the left supraclavicular lymph node (15, 16). The left supraclavicular lymph nodes, often called the Virchow lymph node, is near the junction of the thoracic duct and the left subclavian vein, from which most of the body’s lymph flows into systemic circulation. For breast, cervical, prostate, and even gastrointestinal cancer, lymph diffusion may be along the abovementioned lymphatic drainage routes. Therefore, distant lymph node metastasis of non-solid organs is more contained and has a better prognosis than solid organ metastasis for these malignant tumors.

The diaphragm is an important anatomical structure. It is a natural barrier of the lymphatic system, and it divides the lymphatic system into two regions according to space. Multiple lymph node involvement on one side of the diaphragm has better prognosis than involvement on both sides in regard to Hodgkin’s disease (17). Subphrenic LN (retroperitoneal lymph nodes) are regional lymph nodes in cervical cancer and prostate cancer, which have better prognosis than those with metastasis (18–20).

The nasopharynx is prone to lymph node metastasis because of its well-developed network of lymphatics (21). Yali Xu included 2,994 patients (M1, 299/10.0%) with primary nasopharyngeal carcinoma diagnosed in the SEER database from 2006 to 2015. Compared with the N0/N1 group, the HR of the 5-year overall survival (OS) in the N2 group was 1.311 (95% CI: 1.135–1.514, p < 0.001), and the HR of OS in the N3 group was 1.625 (95% CI: 1.357–1.945, p < 0.01). In addition, the HR of cancer-specific survival (CSS) was 1.351 (95% CI: 1.156–1.580, p < 0.001) in the N2 group and 1.630 (95% CI: 1.342–1.979, p < 0.01) in the N3 group (22).The article gave the tips that the more regional lymph node metastasis in nasopharyngeal carcinoma is, the worse of the prognosis is.

Metachronous metastatic nasopharyngeal carcinoma with different locations has different prognosis. Lujun Shen found that the number of metastatic foci (multiple and single), the number of metastatic sites (multiple and single), liver involvement, and bone involvement were independent prognostic factors of OS, but that distant lymph node metastasis was not associated with overall survival (23). Zixun Zeng and others also analyzed the prognostic factors of 860 patients with metachronous NPC metastasis and found that age, the International Union for Cancer Control (UICC), N stage, Karnofsky Performance Status (KPS), serum lactate dehydrogenase (SLDH), the number of metastases, liver involvement, and bone involvement were prognostic factors affecting the OS of patients with NPC metastasis (24).Jihyun Chang found that distant lung metastasis is a good prognostic factor for metastatic NPC after treatment (25), while most other studies found that heterochronous metastatic NPC with liver metastasis had a poor OS (23, 24, 26, 27).

Distant lymph node NPC metastasis is a common metastasis site besides the liver, bone, lung, and brain (28). In this study, we firstly defined distant lymph nodes as supra- and sub-distant lymph nodes based on the diaphragm and explored the influence of supra- and sub-diaphragmatic distant lymph node metastasis on the prognosis of NPC with metachronous metastasis. Our results showed that subphrenic lymph node metastasis is associated with poorer prognosis. This finding is contrary to the conclusion of the prognosis of distant lymph node metastasis of thoracic, abdominal, and pelvic malignant tumors. It may be that for NPC, distant lymph node metastasis comes from the lymph reflux after the metastasis of peripheral organs. We found that distant lymph nodes were associated with multiple metastasis locations: the mean number of metastatic locations in the group without distant lymph node metastasis was 1.3 (SD, 0.6), and 2.2 (SD, 0.8) in the group with distant lymph node metastasis (p < 0.001). Although metastases of the liver, lung, and supraseptal lymph nodes were not found to predict prognosis in this study, the metastases of subseptal lymph nodes and other organs were found to predict poorer prognosis. However, due to the small sample size of this study, we were unable to detect the impact of liver and lung metastases on prognosis. We speculate that the diaphragm may block the further spread of tumor cells through the lymphatic duct in NPC, which migrates to the surrounding lymph nodes from peripheral organ metastasis (lung, liver, and bone). Once subphrenic lymph node metastasis occurs, it has a worse prognosis than surrounding organ metastasis (liver, subphrenic bone). Yet, supraphrenic lymph nodes do not have a predictive effect for metachronous metastasis NPC due to the better prognosis of the lungs.

While considering from the perspective of molecular mechanism, we may see the other side of SUB-DLN metastases. Lymphatic circulation plays an important role in the occurrence and development of cancer. The dissemination of tumor cells to other organs is usually mediated by lymphatic vessels as catheters, which is often referred to as tumor-associated lymphangiogenesis. When the tumor microenvironment stimulates tumor cells, tumor stromal cells, and tumor-infiltrating cells to induce a series of lymphangiogenic factors, gene lymphangiogenesis related to tumor will occur (29). It was found that miR-129-5p inhibited lymphangiogenesis and lymph node metastasis of nasopharyngeal carcinoma by blocking the zinc finger ZIC2 mediated hedgehog signaling pathway. ZIC2 was highly expressed in nasopharyngeal carcinoma compared with normal tissues. The exogenous expression of miR-129-5p resulted in decreased expression of ZIC2 and other hedgehog signaling components (30). Chuanghua Luo demonstrated that the pigment epithelium-derived factor (PEDF) is lowly expressed in human NPC tissues with poor prognosis and is negatively correlated with lymphatic vessel density (LVD). It was found that PEDF inhibits lymphangiogenesis and lymphatic metastasis of NPC *in vivo* experiments. PEDF also reduced the expression and secretion of vascular endothelial growth factor C (VEGF-C) through the nuclear factor-κB (NF-κB) signaling pathway in NPC cells. Their research showed that PEDF plays a vital role in lymphatic metastasis by targeting both lymphatic endothelial cells and NPC cells (31). The mechanism of nasopharyngeal lymphangiogenesis and lymphatic metastasis needs further exploration, which can explain the influence of different lymph node metastases in NPC on prognosis and also provide a candidate drug for the treatment of NPC metastasis.

This study has several limitations. One, the follow-up treatment of some patients with metastatic NPC was not uniform, and the influence of treatment factors on prognosis was not included in this study. Two, because the EBV DNA level was not available in most of the cases, we cannot analyze its impact on prognosis. Three, only a small number of patients (44/219, 20.1%) were diagnosed with metastases by whole-body 18F FDG PET-CT, and the vast majority of patients were diagnosed *via* chest and abdominal CT; therefore, the incidence of lymph node metastasis may be underestimated. Four, due to the small sample size, the above factors may lead to bias. Further prospective studies are needed to verify the above conclusions.

## Conclusion

Subseptal lymph node metastasis predicts poorer prognosis for NPC patients with metachronous metastasis; however, this needs validation by large prospective studies. This is the first study to divide distant lymph node metastasis into upper and lower parts with the diaphragm as an anatomical boundary. This research provides another perspective and future direction to further explore the relationship between lymph node dissemination and NPC and help us find the treatment for NPC metastasis.

## Data Availability Statement

The raw data supporting the conclusions of this article will be made available by the authors, without undue reservation.

## Author Contributions

D-PC and X-FZ designed the study. YZ, X-WL, J-LC, S-FZ, W-JY, M-YW, and E-LD acquired the data. YZ and X-FZ analyzed the data. X-FZ drafted the manuscript. D-PC critically revised the manuscript’s intellectual content. All authors contributed to the article and approved the submitted version.

## Conflict of Interest

The authors declare that the research was conducted in the absence of any commercial or financial relationships that could be construed as a potential conflict of interest.

## Publisher’s Note

All claims expressed in this article are solely those of the authors and do not necessarily represent those of their affiliated organizations, or those of the publisher, the editors and the reviewers. Any product that may be evaluated in this article, or claim that may be made by its manufacturer, is not guaranteed or endorsed by the publisher.
